# Charting a new course in healthcare: early-stage AI algorithm registration to enhance trust and transparency

**DOI:** 10.1038/s41746-024-01104-w

**Published:** 2024-05-08

**Authors:** Michel E. van Genderen, Davy van de Sande, Lotty Hooft, Andreas Alois Reis, Alexander D. Cornet, Jacobien H. F. Oosterhoff, Björn J. P. van der Ster, Joost Huiskens, Reggie Townsend, Jasper van Bommel, Diederik Gommers, Jeroen van den Hoven

**Affiliations:** 1 Erasmus MC University Medical Center, Department of Adult Intensive Care, Rotterdam, The Netherlands; 2 Julius Center for Health Sciences and Primary Care, University Medical Center Utrecht, Utrecht University, Utrecht, The Netherlands; 3 Department of Research for Health, Division of the Chief Scientist, World Health Organization, Geneva, Switzerland; 4 Section editor Intensive Care at Nederlands Tijdschrift voor Geneeskunde (Dutch Journal of Medicine), Amsterdam, The Netherlands; 5 Department of Intensive Care, Medisch Spectrum Twente, Enschede, The Netherlands; 6 Delft University of Technology, Faculty of Technology, Policy and Management, Delft, The Netherlands; 7 Microsoft, Healthcare, Amsterdam, The Netherlands; 8 Vice President Data Ethics Practice, SAS Worldwide Headquarters, Cary, N.C. USA; 9 National Artificial Intelligence Advisory Committee, Executive Office of the President of the United States, Washington, D.C. USA

**Keywords:** Health care, Medical research

## Abstract

AI holds the potential to transform healthcare, promising improvements in patient care. Yet, realizing this potential is hampered by over-reliance on limited datasets and a lack of transparency in validation processes. To overcome these obstacles, we advocate the creation of a detailed registry for AI algorithms. This registry would document the development, training, and validation of AI models, ensuring scientific integrity and transparency. Additionally, it would serve as a platform for peer review and ethical oversight. By bridging the gap between scientific validation and regulatory approval, such as by the FDA, we aim to enhance the integrity and trustworthiness of AI applications in healthcare.

Fueled by the potential to improve patient outcomes and clinical decision-making, artificial intelligence (AI) is poised to broadly reshape medicine resulting in an exponentially growing number of studies, for example in the field of intensive care medicine^1^. This trend is exemplified by the rapid growth of AI-based trials registered at clinicaltrials.gov. since 2009 — 76 trials from 2009 to 2019, with an additional 294 trials in just the next three years^2^. In 2019, the US Food and Drug Administration (FDA) has launched a digital health branch, approving 692 AI-based models to date^3^. Most of these FDA-approved models, however, are based on evidence from retrospective, single-institution data, often unpublished, rather than robust evidence from clinical trials, the cornerstone of medicine^4,5^.

## Our obligation to ensure responsible AI

AI algorithms are increasingly utilized to assist healthcare providers in clinical decision-making. These AI clinical decision support algorithms derives inputs from various clinical sources, aiding in tasks ranging from classification and computer-aided diagnosis in radiology to clinical prediction models for prognostic or quality purposes^6^. The trustworthiness of such AI algorithms is crucial for their successful integration into clinical practice. In 2020, several authors led an initiative to create an open access database exclusively for FDA approved clinical based AI algorithms^7^. Nonetheless, more detailed reporting is necessary to enhance the understanding and interpretation of AI outputs, thereby fostering user trust and facilitating the integration of AI into a learning healthcare system^8^. The World Health Organization (WHO) has recently published a set of key principles to augment trust in and adoption of AI in health care, including the imperative to improve transparency by detailing the source code, database, data inputs, and analytical approaches used in AI algorithms^9^. While guidelines like SPIRIT-AI^10^, CONSORT-AI^11^, and DECIDE-AI^12^ promote algorithmic information reporting in scientific publications for transparency, they lack specific requirements to translate principles into practice^13^.

To ensure the responsible use of AI algorithms, establishing a supportive infrastructure that builds trust in these systems and mitigates biases during early research, clinical evaluation, and development phases is essential. This concept underpins the European Union’s AI Act, aiming to regulate AI use by addressing potential risks to human life^14^. Thus we advocate for the mandatory registration of early-stage AI algorithms, drawing parallels to the registration of clinical trials.

## Why AI algorithms should be registered

The integrity of clinical trials rests in large part on medical practitioners’ ethical obligation to ensure patient health and well-being, including those involved in research. As the research landscape rapidly evolves, the Declaration of Helsinki is subject to changes to safeguard and maintain trust in research^15^. The Council of Europe’s Helsinki 2019 update conference underscored the need for algorithmic transparency and effective supervisory mechanisms in AI’s design, development, and deployment phases^16^. These measures are necessary to fulfilling ethical obligations, mitigating algorithmic bias, and fostering trust, thereby maximizing benefits and minimizing risks to human rights. AI trials, like human participant studies, must uphold ethical standards, considering the emerging risks of human-AI interactions, interpretability challenges, and data constraints^17^. To ensure a safe translation of AI algorithms into medical practice, it is crucial to understand the design, development, and clinical validation process to infer potential risks of bias and avoiding harm to patients, which would be unethical and could expedite serious negative consequences^11^. Transparency is needed to assess the quality of AI algorithms for stakeholders and to enable medical end-users and patients.

On the other hand, AI algorithm producers (vendors or industry) may be unwilling to provide training datasets or summary information due to intellectual property (IP) and trade secrecy. The intent here is, however, to strike a balance between disclosing algorithm information and protecting IP to promote greater transparency while allowing entities to safeguard their innovations. For instance, enhancing model transparency by disclosing information on model development, training, and validation datasets, and clinical performance is a critical step toward trustworthy AI. This transparency is essential to address AI algorithms’ core components and mitigate potential biases and safety issues. AI algorithm registration should support a dynamic learning healthcare system, allowing for modifications to AI systems post-approval. This iterative design promotes trust and ensures AI algorithm registration aligns with stakeholders’ moral obligation to avoid harm^18,19^.

Currently, the majority of the 14 available CE-certified AI-based radiology products in Europe lack information on training data collection and population characteristics, and none report potential performance limitations related to bias mitigation characteristics, such as ethnicity and age^20^. Both are an obstacle to assess the risk of algorithmic bias. An example is the sepsis prediction algorithm developed by Epic (Epic Systems, WI, USA), which, despite its deployment in several U.S. hospitals, faced poor performance during external validation in 27,697 patients due to a lack of transparent information on performance metrics and the dataset^21^. Early registration could mitigate potential harm by mandating the disclosure of key AI algorithm aspects prior to clinical implementation, encouraging the publication of negative results, and preventing publication bias or overly optimistic interpretations of results. This is exemplified by studies that demonstrated that AI was found to reinforce systematic health disparities^22,23^. Although transparency alone does not ensure bias-free algorithms, it is crucial for identifying and eliminating bias, thereby facilitating continuous improvement and accountability^24^.

## Welcoming AI registration in medicine

The practice of registering clinical trials was initiated decades ago, with the WHO establishing the International Clinical Trials Registry Platform (ICTRP) in 2005 and the World Medical Association’s Declaration of Helsinki mandating prospective registration of all clinical trials since 2008^5,25^. Clinical trial registration has been effective in logging and providing comprehensive information about experimental clinical interventions, significantly enhancing transparency and reducing reporting bias. Similarly, the recent WHO guidance on large multi-modal models encourages the early-stage registration of AI algorithms to improve “explainability,” for instance, by disclosing performance in internal testing^26^. However, current databases like EUDAMED, the FDA database, as well as clinical trial registries, lack fields for early stage algorithm or training data information^20,27^. Given AI algorithms´ potential impact on patient care, traceability and comprehensive documentation of the development process and pre-clinical evaluations are essential. Our proposed set of minimum criteria for an AI algorithm registry aims to fill this gap, requiring registration to encompass the entire model, including data acquisition process details, training data characteristics, model specifications, and information presentation to end-users. (Table 1). This registry does not aim to share code, safeguarding IP, but to ensure that general algorithm information is disclosed, facilitating a safe, transparent, and responsible integration of AI in healthcare. Importantly, the AI system content should not be a concern in terms of patent infringement as only general algorithm information are required. AI algorithms should be registered prior to its deployment in clinical practice and before submitting a trial protocol for ethics approval in preparation for clinical assessment, once the registry is open for enrollment. The registry is designed to capture the lifecycle of AI algorithms in healthcare, recognizing that these models evolve through active learning or subsequent updates with new data. While the focus is initially on the ‘base’ algorithm, the system is intentionally designed to accommodate modifications. The registry should differentiate between minor adjustments, unlikely to impact the AI’s fundamental decision-making process and substantial changes that might affect the model’s performance. Such modifications, including retraining on new data or alterations in algorithmic processing, necessitate updates to the registration. Moreover, for models engaged in active learning or subject to frequent updates, we advocate for a mechanism within the registry that allows for the periodic reporting of updated performance metrics, ensuring the registry accurately reflects each algorithm’s current capabilities and performance in practical applications. The registry’s functional requirements should at least allow for data quality, accessibility, source integration, technical functionality, and governance requirements (Table 2). Specifically because foundation models, i.e. generative AI, such as ChatGPT, released by OpenAI in 2022, differ from well-known general AI models that have the ability to perform specific clinical tasks, such as predicting sepsis^28^. These generative models, characterized by their training on extensive datasets and the utilization of billions of parameters, demand specific hardware and exhibit a dynamic nature. Despite these variances, it’s imperative to trace and log key characteristics to ensure responsible use of AI in clinical decision support^29^. This is much needed because current uses of generative AI within healthcare are limited by their lack of generalizability and limitations of model details, such as model weights, published due to data privacy concerns^27^. Our proposed registry, therefore, distinguishes between generative and general AI in terms of required documentation (Table 1), encompassing training data knowledge corpus of the foundation model (such as time period of training, geographical regions, and languages), implemented policies to prevent the dissemination of sensitive input data into foundation models, details about the manufacturer, and software version.

**Table 1:** Proposed registration information for early stage clinical AI algorithms in healthcare

Item^a^	Description	Generative AI^b^	Non-Generative AI^b^
Name, Version, and AI Model Type	Name of the system, its version, and the type of AI model used (e.g., deep learning, decision tree, etc.)	✓	✓
Training and validation population	Demographics (e.g., age, gender, ethnicity) of the patient population on which the algorithm was trained	✓	✓
Clinical context	Model application (e.g. used for administrative purpose or for instance to predict a specific illness)	✓	✓
Performance Metrics	Performance of the AI system in preclinical development/validation and prior clinical studies (e.g., model discrimination and calibration)	✓	✓
Input Data Types	Types of data used as inputs by the AI system (e.g., images, clinical notes, lab results, etc.)	✓	✓
Data Acquisition and Processing	Process of data acquisition, the steps required for input data entry, the pre-processing procedures applied, and the methodologies employed for handling missing or low-quality data	✓	✓
Output Types and Presentation	Types of outputs generated by the AI system (e.g., predictions, recommendations, etc.) and how these outputs are presented to the users	✓	✓
Registrant Information	Name, affiliation, and contact information of the person or organization that registered the AI system	✓	✓
Foundation model-specific information	Type and version of the foundation model used (e.g., LLM, version: GPT-4 or PaLM 2)	✓	
	Manufacturer or company that developed the foundation model (e.g., OpenAI, Microsoft, Google etc.)	✓	
	Fine-tuning or grounding process used on the foundation model	✓	

*AI* Artificial Intelligence, *LLM* Large Language Model, *GPT-4* Generative Pre-trained Transformer 4, *PaLM 2* Pathways Language Model.

^a^Items to be registered have been adapted from DECIDE-AI and CONSORT-AI guidelines.

^b^The terminology “Generative AI” and “Non-Generative AI” specifies if certain data or criteria are relevant to generative AI models, non-generative AI models, or both. The presence of a checkmark (✓) in a column signals that the mentioned data or criteria pertain to that AI category. Generative AI models are capable of producing new content, such as the way Large Language Models (LLMs) can craft text that mimics human writing. On the other hand, Non-Generative AI models are designed to interpret and learn from pre-existing data to make forecasts or decisions. For instance, these models analyze and learn from existing data to make predictions or decisions.

**Table 2:** Minimal functional requirements for the registry

Requirement	Description
Data quality	‐ Verification mechanism for registration data accuracy
	‐ Assignment of unique identifiers to distinct algorithms
	‐ Prevention mechanism for duplicate algorithm registrations
	‐ Accessible audit trail and version management
Accessibility	- Accessible to all potential registrants
	- Freely available to the public
	- User-friendly for contributions
	- Electronically searchable
	- Offered in relevant languages
	- Open 24/7 for submissions and searches
Integration with other sources	- Enable linking with clinical trial identifiers
	- Enable linking with published study DOIs
	- Enable linking with FDA or European Commission device identifiers
	- Automate data transfer between third parties, reducing redundancy
Technical functionality	- Routine maintenance and updates
	- Ensure permanence of entries
	- Efficient data storage and management
	- Robust data protection against loss and corruption
Governance	- Central global hub for data entry, reducing redundancy
	- Administered by a non-profit organization like NIH’s clinicaltrials.gov

*AI* Artificial Intelligence, *NIH* National Institutes of Health, *FDA* Food and Drug Administration, *DOI* Digital Object Identifier.

Institutional review boards should consider algorithm registration a prerequisite for approval, and scientific journals could make registration a condition for publication, continuing a tradition of rigorous scientific accountability, as has been done in the past^5^. Healthcare institutions should consider the prerequisite of early registration, fostering a culture of transparency, even in situations not subject to regulatory or other oversight. Such proactive measures act as a safeguard against the deployment of unverified algorithms that might endanger patient safety. Integrating algorithm registration into the current practice could ensure the safe, transparent, and responsible integration of AI in healthcare. While early registration will foster transparency, accountability, and eventually ensure patient safety, it is imperative to strike a balance between capturing knowledge at an early stage and minimizing registration burden. We therefore advocate for an iterative and flexible registration process that can adapt to the evolving landscape of AI in healthcare. AI registration represents a crucial important advancement to improve safety and responsible use of AI in healthcare. It responds to the growing ask and need for regulatory frameworks, regulatory oversight and robust solutions^27,30^.We encourage governmental agencies, national and international organizations, AI experts, and the private sector (including tech companies) to bundle forces and knowledge to facilitate and regulate such a registry.
